# Next-generation sequencing yields the complete mitochondrial genome of *Pterygoplichthys pardalis* (Loricariidae; Siluriformes)

**DOI:** 10.1080/23802359.2021.1959447

**Published:** 2021-10-20

**Authors:** Wangxiao Xia, Hui Jiang, Jinghao Ge, Jianghong Cheng, Peng Liu, Xingchun Gou

**Affiliations:** a Shaanxi Key Laboratory of Brain Disorders, Institute of Basic Translational Medicine, Xi’an Medical University, Xi’an, China; b National Engineering Laboratory of Marine Germplasm Resources Exploration and Utilization, Zhejiang Ocean University, Zhoushan, China

**Keywords:** Catfish, Loricarioidei, mitogenome, molecular data, phylogeny

## Abstract

In this study, we report the complete mitochondrial genome of *Pterygoplichthys pardalis* has derived by next-generation sequencing. The complete mitochondrial genome of *P. pardalis* contains 16,425 bp encompassing 13 protein-coding genes, 22 transfer RNA genes, two ribosomal RNA genes, and one control region (D-loop). The base composition is A 31.79%, C 26.89%, G 14.63%, and T 26.69%, and its gene arrangement is consistent with mitochondrial genomes derived from other representatives of Loricariidae. A phylogenetic tree of 24 Loricariidae species constructed based on the 13 coding genes shows that *P. pardalis* is clustered with other *Pterygoplichthys* genus. It suggests that the molecular classification results confirm its external morphological characteristics. These results have reference value for the further study of phylogenetic relationship, taxonomic classification, and phylogeography of Loricariidae.

*Pterygoplichthys pardalis* (Castelnau, 1855) is an neotropical sucker mouth armored catfish, that is extremely exported as ornamental aquarium fish to many other countries and often released becoming an invasive species (Nico et al. 2009; Ebenstein et al. 2015; Lujan et al. 2015; Kumar et al. 2018). Due to its strong survival ability and rapid spread, it has caused damage to the local ecological environment (Nico et al. 2009; German et al. 2010; Jumawan et al. 2010; Lujan et al. 2015; Nurubhasha et al. 2019). As a relatively well known representative species in Loricariidae, *P. pardalis* has long been the subject of intense interest among ecological biologists (Hoover et al. 2004; Chaichana and Jongphadungkiet 2012). However, its complete mitochondrial genome was not known until now.

To sequence, assemble, and annotate the complete mitogenome of *P. pardalis*, one individual was acquired from the Chinese breeding base in Xi’an city (108°41′E, 33°85′N) and deposited at the lab of Institute of Basic Translational Medicine, Xi’an Medical University under the voucher number 20190815PP01. The species identification was determined from the sequence of the molecular data and multiple morphological features, such as the characteristics of head (radiate pattern of light lines) and body (hexagonal leopard patches) (Rao and Sunchu 2017). Genomic DNA of *P. pardalis* was extracted from muscle tissue using Qiagen Blood & Cell Culture DNA Mini Kit. Then, the short paired-end library was constructed by Illumina protocol and sequenced with HiSeq X-Ten PE150 platform. MitoZ software was applied to assemble the mitogenome with default parameters (Meng et al. 2019). The mitochondrial genes were predicted and annotated using the MITOS Web Server (Bernt et al. 2013) and tRNAscan-SE Search Server (Chan and Lowe 2019). Finally, the assembly and annotation results of the complete mitochondrial genome of *P. pardalis* were made publicly accessible (GenBank accession number: MW401674).

The whole mitogenome sequence of *P. pardalis* has 16,425 bp in length, containing 13 protein-coding genes, 22 tRNA genes, two rRNA genes, and one control region (D-loop). The base content and gene arrangement were similar to other species of teleosts (Jiang et al. 2017, 2018; Li et al. 2019; Liu et al. 2019a, 2019b). Almost all the protein-coding genes were encoded by H-strand except *ND6* and eight tRNAs (Gln, Ala, Asn, Cys, Tyr, Ser, Glu, Pro) genes which are located on the L-strand. In addition, 12 protein-coding genes start with the start codon ATG, while *COX1* use GTG as the start codon. For the stop codon, six protein-coding genes (*ATP6*, *ATP8*, *COX1*, *ND1*, *ND4L*, *ND5*) end with TAA, only *ND2* and *ND6* ends with TAG, and *COX2*, *COX3*, *CYTB*, *ND3*, and *ND4* ends with T. The base compositions are A 31.79%, C 26.89%, G 14.63%, T 26.69%, and AT and GC contents are 58.48% and 41.52% with a significant AT bias, respectively. The 13 protein coding genes are 11,385 bp, accounting for 69.31% of the whole mitogenome, which encodes 3795 amino acids in total. The predicted lengths of 12S rRNA located between tRNA^Phe^ and tRNA^Val^ and 16S rRNA located between tRNA^Val^ and tRNA^Leu^ are 955 bp and 1686 bp, respectively. The control region located between tRNA^Pro^ and tRNA^Phe^ is 786 bp.

To phylogenetically infer the position of *P. pardalis* within Loricariidae with available mitogenomes, the sequence alignments were performed by ClustalW (Thompson et al. 1994) in BioEdit software (Hall 1999) and the phylogenetic tree was constructed in Mega7 by the neighbor-joining (NJ) method with 10,000 bootstraps (Kumar et al. 2016). The phylogenetic relationship of *P. pardalis* and other 23 Loricariidae species (from six different subfamilies) were analyzed based on the 13 protein-coding genes with *Sternopygus dariensis* Meek & Hildebrand 1916 (Gymnotiformes) as the outgroup. The result shows that *P. pardalis* is clustered with other *Pterygoplichthys* genus. Our result confirms that the identification based on morphological features corroborates with the molecular analysis (Figure 1). We expect that these results would provide valuable reference data for other studies.

**Figure 1.**
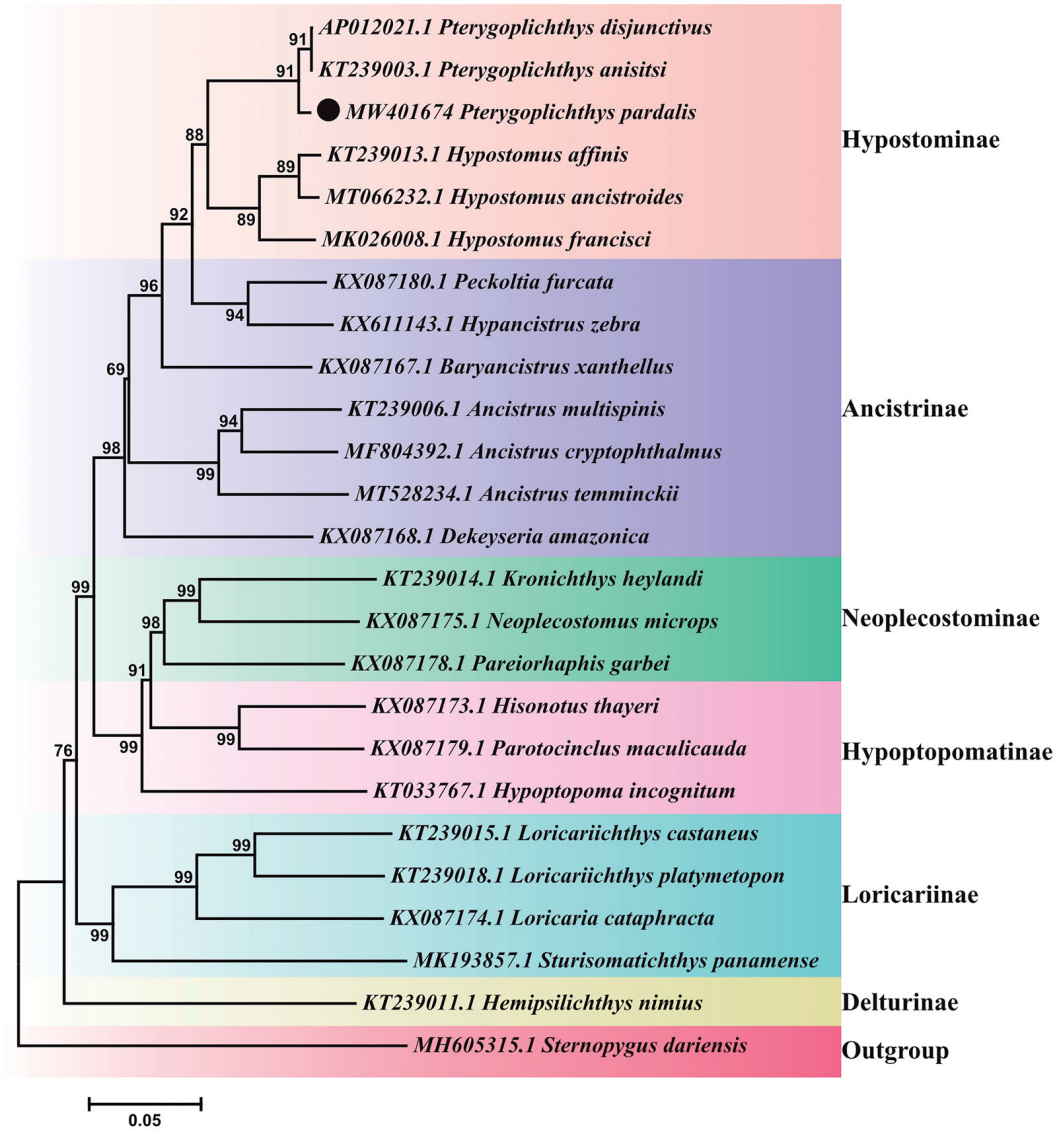
Neighbor-joining tree of 24 Loricariidae and one outgroup (*Sternopygus dariensis*) species based on the 13 protein-coding genes. The number at each node is the bootstrap probability. The number before the species name is the GenBank accession number. The dark spot indicates the species in this study.

## Data Availability

The data that support the findings of this study are openly available in NCBI at https://www.ncbi.nlm.nih.gov/, reference number [MW401674], or available from the corresponding author.
